# Reviewing School Uniform through a Public Health Lens: Evidence about the Impacts of School Uniform on Education and Health

**DOI:** 10.3389/phrs.2021.1604212

**Published:** 2021-06-16

**Authors:** Johanna Reidy

**Affiliations:** Department of Public Health, Wellington School of Medicine, University of Otago, Wellington, New Zealand

**Keywords:** school uniform, public health, equity, health impacts, education impacts, human rights

## Abstract

This study uses a public health lens to review evidence about the impacts of wearing a school uniform on students’ health and educational outcomes. It also reviews the underlying rationales for school uniform use, exploring historical reasons for uniform use, as well as how questions of equity, human rights, and the status of children as a vulnerable group are played out in debates over school uniforms. The literature identified indicates that uniforms have no direct impact on academic performance, yet directly impact physical and psychological health. Girls, ethnic and religious minorities, gender-diverse students and poorer students suffer harm disproportionately from poorly designed uniform policies and garments that do not suit their physical and socio-cultural needs. Paradoxically, for some students, uniform creates a barrier to education that it was originally instituted to remedy. The article shows that public health offers a new perspective on and contribution to debates and rationales for school uniform use. This review lays out the research landscape on school uniform and highlights areas for further research.

## Background

Despite regular judicial, community, and press scrutiny, there is little consensus on the function of school uniforms, or agreement about evidence of their impact on education and health. Breaches of school uniform policy have resulted in court cases (e.g., [1, 2]), and courts note that in focusing on the rights and wrongs of a particular uniform policy, the underlying issues driving uniform design and policy are neglected [3]. Meanwhile, at the beginning of the school year in many English-speaking countries there are numerous press articles about the cost burden to families of providing school uniforms [4–8], whether they are value for money [9–11], and whether garment design is fit for modern life [12–17]. Discussion seems stymied in a superficial argument about whether school uniforms are good or bad. Rarely do discussions point to empirical evidence about school uniform garment design and policy about uniform use. This situation begs questions as to availability of evidence for school uniform use, its effects on educational or health outcomes, and the underlying rationales for school uniform use.

This article applies a public health lens to review evidence about why we have uniforms and what effects they have on educational and health outcomes. A public health perspective was chosen to review evidence because it is explicitly designed to analyze impacts of broad socio-political forces and determinants of health on individual experiences. Further, public health sees education and health as mutually reinforcing and intrinsically linked. The one determines the success of the other. Consequently, much public health policy aims to optimize wider social policy settings to improve health and education [18], and encourage equitable outcomes especially for the most vulnerable populations [19]. It is also why the World Health Organization (WHO) promotes health in all government policies to improve overall population health ([20]). Therefore, attention to students’ physical and psychosocial health and wellbeing is important for enhancing educational outcomes. This includes evidence for the choice of school uniform garments and individual schools’ policy about uniform and how these affect student wellbeing. The evidence considered here suggests that uniform is of public health concern because its use and effects are prevalent, have impact and are amenable to improvement. Uniform use is prevalent and widespread globally. In their study of 39 PISA countries, Baumann and Kriskova [21] identify five main geographic/sociocultural groupings where uniform wearing is common: an Anglo-Saxon cluster (United Kingdom, NZ, Australia, United States), Asia, East Asia (South Korea, Japan), the Americas (e.g., Mexico), and Europe. These authors also note that uniform prevalence is increasing. Regarding impact, evidence shows uniforms can impact directly and indirectly on the individual and on society in equity, health and educational domains for better and for worse. The reviewed literature suggests that any harms are amenable to intervention *via* evidence-based action. Meadmore and Symes [22] argue that uniforms are not as frivolous as they appear and warrant systematic attention. This article applies that systematic attention through a public health lens. It explores three questions: What is the evidence for the impact of school uniform on students’ academic and health outcomes; what social, cultural and political rationales are made for uniform use; and what human rights may be affected by school uniform choice? For conciseness, “school uniform(s) garments” will be referred to as uniform(s). The practice of wearing/using/mandating a school uniform will be referred to as uniform policy.

## Main Text

### Methods

Databases that include health and education research were searched for peer-reviewed articles in English using the key word “school uniform” in the title keywords or abstract. The date range searched was from 2000 to (present), being October 2020. The results are detailed in Table 1.

**TABLE 1:** Database searches October 2020.

Host	Databases	Limits	Search terms	Number of articles
Ovid	Ovid medline R (ALL) EBM reviews (cochrane database of systematic reviews). ERIC. Philosopher’s index. APA psychinfo	2000-Present (Oct 2020)	School uniform	83
			School uniform AND education	12
			School uniform AND health	36
Scopus		In article, abstract or keywords. 2000- present. Article or review	School uniform	138
			School uniform AND health	20
			School uniform AND education	38
Web of science	All databases	English. Article. 2000–2020	School uniform	45
			School uniform AND health	11
			School uniform AND education	0
EBSCO online database collection^a^	Academic search complete. Australia/NZ references center. Education resource complex. Psychology and behavioral sciences complete	2000-Oct 2020; all; abstract. All. Academic journal; English, abstract. Abstract	School uniform	2
			School uniform AND health	6
			School uniform AND education	0
			Total found	304

a Each database has different possible limits and the platform generates one result.

Oft -cited peer-reviewed sources that did not appear in the literature searches were also included in the literature review (*n* = 25), as well as texts that were found in the initial work for this review. Texts were de-duplicated, yielding 197 texts. Records were screened for relevance and excluded 79 for being out of scope because of time constraints (not in English, PhD theses, conference proceedings). This yielded 118 full text articles to be assessed, of which 26 were excluded because they were off-topic for this review (e.g., industry information about supply chains; school uniform as a basis for a thought experiment; fetishism; reports on forensics; technical information about fabric properties). 92 studies were included in this review.

Note this study examines the breadth of evidence for uniform wearing. Study quality was not part of the analysis.

Articles fell into three broad groups: surveys/studies that elicited stakeholder feedback on some aspect of garment design or policy; or experience of uniform wearing; analyses of large datasets or administrative data; and political, philosophical/ethnographic, and legal analyses of rationale and impact of uniform use.

The first group comprised empirical research that examined data on some aspect of garment design or policy or uniform wearing experience. There was a mixture purposive samples and convenience samples. Studies varied in the number of participants, the number of sites from which participants were taken. Studies elicited views from stakeholders: students, parents, teachers, administrators, social workers, school counselor. Views were gathered *via* survey and/or focus group. Some surveys formed part of a case study. There were also stand-alone case studies and ethnographies, an RCT and an auto-ethnography.

12 studies examined garment properties for Sun protection, safety, design. The mix of stakeholders varied: students only (*n* = 15); students and family/parents/caregivers (*n* = 8); multiple stakeholders (students, parents, teachers, and administrators, and/or social workers) (*n* = 17). There were three randomized control trials. There were a mixture purposive samples and convenience samples. Studies varied in the number of participants, the number of sites from which participants were taken. The second group comprised analyses of large datasets (*n* = 5), and one meta analysis on factors affecting educational outcomes. The third group were non-empirical studies. They included: policy summaries; legal analyses; historical commentaries on uniform’s development; socio-political analyses; political think-pieces; and one economic analysis.

## Results

Here, evidence has been arranged according to a public health lens of analysis. First, this section examines the proximate educational and health impacts of uniform garments and uniform policy on students to determine whether there are immediate health or education impacts of uniform use or policy. Second, rationales for uniform use are examined, as well as distal factors that influence student experience. This section examines the broader institutional, and socio-cultural contexts which inform uniform use.

### Part 1: Literature for Educational and Health Impacts of Uniform

#### Does Uniform Influence Educational Outcomes?

**FIGURE 1:**
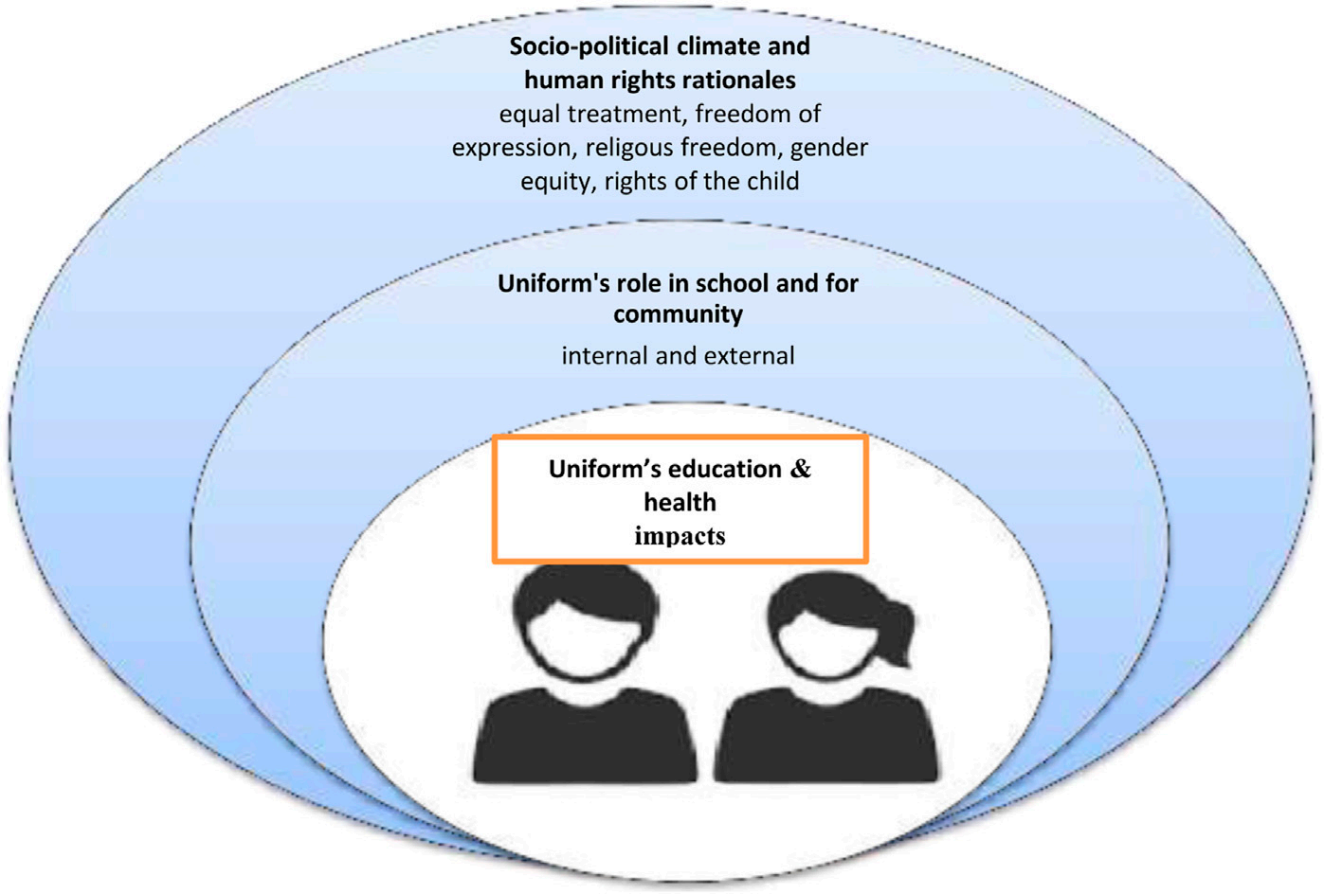
Organization of evidence about uniform use.

Starting with the evidence for the impact of uniform on educational outcomes (the core in Figure 1), there is little convincing evidence that uniform improves academic achievement. Studies from the United States in the early 2000’s [23, 24] note a positive correlation between uniform wearing and academic achievement (e.g., Bodine [25]). Later, in 2012 Gentile and Ibermann found a positive effect on grades and retention [26, 27]. Stockton et al. [28] noted there was a greater *perception* of increased attendance and achievement after uniform was introduced. However, studies of large datasets and meta-analyses fail to find a link between uniform and academic achievement. Brunsma and Rockquemore’s (2003) response to Bodine’s assessment of their administrative data review in the late 1990’s reiterated that no overwhelming link exists between uniform wearing and academic outcomes (there were methodological disagreements about which data to choose and how they should be analyzed). Later studies by Yeung [29] and Creasy and Corby [30] noted multiple factors for academic achievement—but not uniform. In a synthesis of 800 meta-analyses on effects of all hitherto published variables of educational outcomes, Hattie [31] demonstrated negligible to no association between uniform and academic achievement itself. However, he notes that the ‘heat and impact of the discussion are as if [uniform] were obviously effective’ (p106) [32]. In a 2017 update to that study uniform was not listed among the 252 effects on educational outcomes [33].

Nonetheless, it appears that uniform may contribute to an environment that fosters academic achievement. Baumann and Kriskova [21] examined information from the PISA study on student experience of discipline within the classroom environment (listening, noise level, quietening/settling, schoolwork, starting work). This study involved a very large sample of students from across the globe. These researchers found a statistically significant difference related to settling to work between uniform wearing and non-uniform wearing samples. Thus, Baumann and Kriskova [21] recommend keeping uniforms where already used and introducing them where not used. Similarly, Firmin et al. [34] found introducing uniform reduced distractions. Writing about the United States, DaCosta’s [35] study of students noted improved concentration and increased security in the school where uniform was introduced. A South African study reported that uniform helped to maintain classroom discipline [36].

However, settling to work and classroom discipline are two of many facilitators of learning outcomes [21], along with class size, funding levels, homework, and, importantly, factors related to the quality of the teacher (qualifications, personality, incentives, mentoring for new teachers). Given that teacher skill and relationship between student and teacher are established as influential factors on learning outcomes [33], some argue that expecting teachers to enforce school uniform rules detracts from teaching, learning, and good relationships [30, 37], notwithstanding the classroom management benefits of uniform-wearing described by Baumann and Kriskova [21]. Indeed, Da Costa [35] reports, the introduction of school uniform created opposition and non-compliance, distracting students and teachers from education. There are indications that uniform could create psychological barriers to education for vulnerable students, especially when it is a new phenomenon. Gromova and Hayrutdinova [38] found that for ethnic-minority newcomers to a school, uniform can simply be another strange element to get used to in a new environment.

One study argues that organisational and classroom management enhanced by uniforms may be achieved at the expense of other educational goals and values. Baumann and Kriskova’s [21] research ranks Korea and Japan highest in terms of settling to work and removing distractions. Yet Park’s [39] study found in Korea uniform was linked to stifling creativity, in spite of good academic performance. This is indicative only (a small study from one country), but highlights how much is not known about the impact of uniform on other domains of education.

Another effect of school uniform is that schools socialize students to certain explicit and implicit values and social norms and inculcate social skills that will help them get on in the world. Within that framework, school uniform provides what Vopat [40] describes as teachable moments (unplanned, yet important learning opportunities) to reflect on norms of society. There is no data that directly addresses non-academic learning outcomes from uniform. However, Vopat’s idea of teachable moments hints at why some administrators prefer a uniform [41, 42], and a more formal one at that [41].

In some contexts, uniform is also instrumental to other goals: school security and students’ physical safety, aids student focus on learning. In South Africa, Wilken and van Aardt [36] observed that uniforms can make certain students targets of attack outside the school grounds. In South Africa and the United States uniforms are used to easily identify intruders on school premises and to reduce gang violence and theft of designer items outside of school [35, 36]. However, in the United States one study found negligible evidence of uniform enhancing security [43], while another study found introducing uniform created only a lower *perception* of gang presence [44].

Overall, it appears that while uniform is a factor that removes distractions from classroom learning, thereby enhancing operational management, it has no direct impact on academic achievement and is not among factors that demonstrably improve educational outcomes. It may enhance school security, and influence schools’ broader educational and socialization goals.

#### Does Uniform Influence Health Outcomes?

Unlike for educational outcomes, there is a far more direct link between uniform garments and uniform policy and health outcomes. Health impacts can be divided into physical and psycho-social effects, though there is a significant overlap between the two. Physical impacts of school uniform relate to how uniforms facilitate physical activity during the day, whether uniform garments protect the wearer against known environmental hazards, whether the garments promote health and safety, and whether the garments are comfortable to wear. Psycho-social impacts are linked to fitting in (or not) with peers.

One effect uniforms have on physical wellbeing is their limitation or allowance of exercise. Encouraging regular physical activity is part of the WHO’s health promotion concept of health in all policies and settings. Globally, governments are trying increase physical activity among children and young people to reduce child obesity rates [45]. Additionally, physical activity enhances learning outcomes and improves wellbeing ([46]), therefore policies that promote planned and incidental physical activity positively influence educational and health outcomes. However, it appears that school uniform design and policy can pose a barrier to incidental exercise, particularly for girls. McCarthy et al. [47] found primary school girls were more active on sports uniform days and met government recommended daily physical activity levels on those days. Norrish et al.’s [48] study on the effect of uniform on incidental physical activity among ten-year-olds found that school uniform design could limit physical activity (measured by student self-report and pedometers). Correcting for choice of activity (ballgames, skipping vs imaginary play, verbal games), girls did significantly more activity during breaks on sports uniform days. Likewise, Watson et al. [49] and Stanley et al. [50] reported that recommended physical activity for school-aged children was not being met, especially for girls, where restrictive school uniform limited physical activity and created an explicit barrier to lunchtime play. Further, in an age of active transport policy, Hopkins et al. [51] found that school uniform style and lack of warmth was a barrier to cycling to school for some female secondary students, and Ward et al. [52] found both garment design and schools’ uniform policy hampered active transport among older teenagers. There are strong indications that uniform garments and policy about which garments can be worn directly impact on students’ physical health outcomes, for female students in particular.

While there is evidence on how uniform facilitates physical activity, there is little evidence on the psychological effects of uniforms on how students feel about doing physical activity in uniform. Unflattering or revealing (sports) uniforms may deter students from participating in sport. Focusing on physical activity, Watson’s et al.’s [49] study noted the complex social factors that affect physical activity, and how a unisex sport uniform could enhance the feeling of comfort and confidence. For instance, Pausé’s [53] auto-ethnography highlights the psychological barrier an unflattering sports uniform can pose to fat children’s participation in and enjoyment of physical activity as a good in itself (as opposed to a means to lose weight).

Physical health can be protected against known environmental health hazards by uniform garment design and policy implementation. However, school uniform policy (at national or school level) does not routinely address these hazards. In Australasia, ozone layer degradation results in high UV radiation levels in warmer months. Prolonged UV exposure results in skin damage and over the long term increased rates of moles and skin cancers across the population. Yet Gage et al. [54] found that uniformed schools had lower total body coverage than non-uniformed schools, albeit with greater neck coverage due to collared uniforms. This is despite evidence that hats with a brim and sun-safe clothing (covered arms and legs) can improve sun protection [55] while not increasing objective measures of body temperature [56]. Indeed, modeling from Australia indicates that slightly longer garments significantly alter mole patterns [57]. Of course the effectiveness of uniform garments (or indeed any garments) for sun protection depends on proper implementation of policy. For instance, in New Zealand Sunsmart is a voluntary school policy to optimize protection of children’s skin from sun damage and sunburn. However, Reeder et al. [58] found that Sunsmart policies were not consistently implemented, even among Sunsmart-accredited schools.

Uniform has also been used as part of measures to combat disease. In Thailand and other countries with endemic dengue, school uniform design, the use of insecticide-treated clothing [59–62], and how uniform is worn [63] have been investigated extensively in relation to dengue prevention, especially how to stop insecticide washing out of fabric. However, while the use of insecticide-treated clothing is supported by parents in these countries, willingness to pay for the uniform is linked to parental monthly income. Governmental willingness to subsidize treated uniforms is linked to overall cost, irrespective of effectiveness or potential health gain [64, 65]. It appears that good garment design that protects against environmental hazards cannot be separated from good policy implementation and a financial subsidy if garment cost is high.

Interestingly, while environmental hazards and their impact on health were considered, no peer reviewed articles were found related to safe garment design e.g., Inflammable materials, removing strangling risks. The only information found on uniform policy and garment safety did not relate to garments but accessories (not uniform proper). It was from the United Kingdom, where the Health and Safety Executive found that schools had incorrectly applied health and safety legislation to ban certain non-uniform items of jewellery that had no link to causing physical harm [66].

Is it possible to achieve optimal uniform garment design? Researchers have examined different elements of uniform design, some related to health outcomes. There is a particularly interesting body of research emerging about properties of school uniform garments. Researchers have investigated how to standardize sizing [67], improve garment quality and durability [68], optimize materials, enhance style, include high visibility/reflectiveness for road safety, and ensure physical comfort irrespective of outside temperature [68–71]. This demonstrates that it is technically possible to design a uniform that meets cost imperatives, is physically safe, comfortable, and enjoyable to wear. These studies showed garment materials do not necessarily prioritize the wearer’s physical comfort. Functionality (durability, ease of care, ease of drying, stain and wrinkle resistance) is often preferred over comfort or safety (Kadolph, 2001 in 36). For example, polycotton is used instead of cotton because it is colourfast and fast-drying, despite not breathing well in hot weather.

It appears that no consensus exists on best practice for uniform design, who should be involved in design decisions, and considerations in policy development and implementation (e.g., health and educational impacts of garment design and policy, gender neutral options, non-physically restrictive garments). There is no data that discusses this point directly though some studies involve parents and students [68, 71], and DaCosta [35] recommends involving students in co-designing the uniform, to develop a uniform that provides choice and flexibility. Gereluk proposes principles for a non-discriminatory environment [72], which provides helpful guidance on how to accommodate minority concerns into majority spaces. In doing so, he helpfully lists general elements to consider that can be applied to uniform design and policy. These are: health and safety; whether (any religious/cultural garment) is oppressive to (the wearer) or others; whether it significantly inhibits the educational aims of the school; whether (whatever item is not part of the uniform) is essential to one’s identity.

There is evidence that uniforms can be psychosocially protective of health. Uniforms remove “competitive dressing”—the pressure to wear certain (expensive) brands, colors, or styles [36]. Uniform removes most socio-economic signs of difference [73]. Wilken and van Aardt [36] and Jones (for higher socio economic status students) [74] report that school uniforms take away stress and family arguments about what to wear on school days. The positive psychological effect of removing competitive dressing probably only holds for students with a certain level of material wealth (see discussion below on equity of access to education and uniform cost). Thus, Catherine and Mulgalavi [75] found in Pakistan that school uniform had a positive effect on students’ self-esteem, particularly if they had the full and correct uniform. It seems for very poor students, school uniform requirements may simply become something else to worry about, but for others uniform removes a barrier to fitting in.

In addition to the ambivalence of wearers’ feelings, there are mixed data on the impact of uniform on bullying. In a study of one school in the United States, Sanchez et al. [76] found introduction of a uniform did not significantly change the school’s culture before and after a school uniform was introduced, though some females said males treated them better when they wore a uniform. Jones (United States) reported a reduction in bullying after uniform was introduced [74].

Indeed, Cunningham and Cunningham [77] note that while uniforms can reduce bullying, there will always be triggers such as girls choosing to wear trousers not skirts. Importantly, any dress is about more than clothing, indicating social relations, self-presentation, and formation in society, and is a sensitive topic in adolescence [78]. Indeed, Swain’s ethnography found that students who complied with uniform rules risked being socially excluded [79].

It appears that uniforms can be both protective and harmful, depending on context, how the student pushes the boundaries of uniform rules to fit in, and whether the student is part of a marginalised/socially disadvantaged group. Whatever the context, females are half of the population, and their physical and psycho-social health seems to be routinely and arbitrarily disadvantaged by uniform design.

Overall, in terms of health and education impacts it seems any psycho-social benefits will only hold if other psycho-social and physical harms to girls, and minorities are addressed. Table 2 summarizes the health and education impacts of uniform. From a health and education perspective, uniform’s biggest advantage is that it removes some distractions; it helps students to settle in the classroom and removes the worst of competitive dressing. If garments and policy are well designed, they encourage physical activity and can protect against environmental hazards. Nonetheless, poorly designed garments and uniform policies especially affect girls and minorities.

**TABLE 2:** Uniform’s positive, neutral, and negative impacts on education and health outcomes.

Domain	Positive impact	Neutral impact	Negative impact
Educational outcome	Improves classroom management; faster settling to task; reduced distractions	No clear impact on academic achievement	Detracts/distracts from teacher–student rapport; Reduces creativity in population; An extra barrier to socialisation for newcomers/minorities
Health outcomes: Physical	Insecticide-treated uniform can provide protection against dengue; Well-designed uniform can protect skin from sun damage; Health and safety can be enhanced in uniform design		Poor uniform policy a barrier to incidental and curriculum based exercise; Poor uniform design a barrier for incidental exercise, especially for girls; Sun protection not considered in policy or garment design; Physical comfort/health not prioritised in design; Health and safety can be misapplied to ban certain non-uniform items
Health outcomes: Psychosocial	Unisex and inclusive design can increase girls’ and overweight students’ confidence to participate in sport; Self-esteem promoted (if student can afford full and correct uniform); Removes competitive dressing pressure; Decreases bullying		Non-inclusive design can reduce girls’ and overweight students’ confidence to participate in sport; Bullying and social exclusion for following uniform rules; Inflexible uniform policy harmful for gender-diverse students

### Part 2: Exploring Social, Cultural and Political Rationales for Uniform Use

Since uniforms do not positively influence academic achievement and can have negative physical and psycho-social health impacts, what drives their use? Further, why are known problems in uniform policy and design not addressed? To answer these questions, it is important to consider the broader context in which uniform is used. The literature that addresses these questions can be divided into three groups. The first group examines the role of uniforms in institutions and the community; the second, the interaction between human rights and uniform; the third (dealt with in part 3 below) the relationship of uniforms to the idea of children as a vulnerable class of people who need special protection. Institutions, human rights laws and societal perceptions of children and childhood constitute important upstream/distal determinants of health and educational outcomes. All the above elements contribute to wider social settings that facilitate or prevent access to what people need to enjoy good health and education. Table 3 summarizes rationales for uniform use.

**TABLE 3:** Implicit and explicit rationales for uniform use.

Rationales for use from literature
School culture/operational management
Symbolic of school culture; encourages pride
Affiliation supported by sameness
Delineates social hierarchies (in/out groups)
Improves school security/perception of school security
Removes socio-economic differences between students
School’s reputation/impression management *via* student appearance; well-disciplined body of students
Signals school’s place in education market
Socio-political context
Social mores—garment design and rules change over time reflecting societal change
Historically as a transfer of values—especially in post-colonial anglosphere
Currently auxiliary to achieving wider public policy goals: Cultural revitalization, introduction of sharia laws, enhancing feeling of citizenship/patriotism
Instrumental—tool to equalize social class in school *via* social camouflage
Encourages social integration and cohesion
Human rights expressed in context
Equality**–**Enhances access to education
Freedom of religion
Minorities bear burden of sameness and make more accommodation to uniform
Uniform policies sometimes do not/cannot legally accommodate difference
Gender equality
Restrictions based on historical norms
Girls’ movement restricted by design
Girls’ uniform garments more expensive than boys’
Sex vs. gender: Gender non-binary students discriminated against by uniform policy
Freedom of expression
Hampers expression vs. there being many other outlets for student expression/students can rebel in safe confines of uniform
Right to expression linked to age and stage of development
Provides teachable moments about social attire, appropriateness
Fosters suspicion of other/difference
Reduces ability to discuss difference
Children as a vulnerable group
Unclear what aspects of childhood/children need protecting according to age and developmental stage
Unclear whether uniform should be imposed when it does not positively affect academic achievement
Duty to consider children’s/youth voices in garment design and uniform policy according to age and stage of development

#### Uniforms as a Reflection of Schools and Communities

Schools are institutional extensions of overlapping communities: geographic, religious, or ethnic. Community norms reflect institutional and wider societal rules. Uniform signals internal culture to students and provides cues to outsiders about the school’s character.

Within schools, uniforms reinforce institutional culture, signaling school values to students [80], thereby identifying the wearer with objectives beyond the self. Along with school facilities and symbols [21], a well-disciplined body of students is associated with a certain type of dress. Additionally, some argue that uniforms contribute to a sense of affiliation in students, belonging [81], and pride in the school, especially after uniform has been recently introduced [82]. Affiliation is related to solidarity; yet there seems to be a tipping point when solidarity is undermined if the uniform is too expensive and excludes students [83]. Howell [84] argues that among charter school students he studied in the United States, uniform is only one element to increase participation and is far less important than other variables like family dynamics. However, claims about uniform fostering solidarity are not supported by empirical research on student feelings about belonging in the school context. Research into school belonging did not find a significant association between school uniform and a sense of belonging to the school community [85]. Instead, belonging is fostered by a supportive, respectful atmosphere and a sense of achieving.

It has been argued that uniforms communicate messages to those outside the school community. Stephenson [86] argues the main role of uniform has changed from primarily addressing poverty or removing differences marking class and gender to primarily signaling education standards, and the school’s place in the education market [22, 36], showcasing the institutions’ disciplinary philosophy [27]. Happell [87] notes that in the United States uniform visually demarcates students and is associated with private education, improving the wider school environment [35], or maintaining the impression of strictness and safety [22]. Shao et al. [88] note that like corporate uniform, school uniform gives cues to the service environment—a more conservative uniform suggests more conservative values, higher socio-economic status, and by association higher academic achievement. Indeed, Bodine [89] notes that uniform reinforces and delineates social hierarchies and who belongs. Belonging can be inclusive, encouraging broad participation and access, or exclusive by drawing lines between people and putting up practical barriers to access, delineating who is and is not worthy of privilege [90].

Within institutions uniform is a management tool [21]. It has the veneer of solidarity, but there is no empirical evidence linking uniform to feelings of belonging to a school. Uniform also signals tradition, and communicates the place in the education market to outsiders, especially a school’s disciplinary and academic climate. The factors affecting a school’s choice to require a uniform is in turn affected by wider forces of socio-political climate and human rights.

#### Wider Forces: Socio-Political Climate

As illustrated in Figure 1, the individual health and educational impacts of uniform are nestled in the broader school culture, which in turn is influenced by the wider socio-political context, influenced by the community’s values. A country’s history, power structures, and socio-economic patterns are thus played out through uniforms. Further, dominant societal values are the lens through which human rights and other implicit and explicit values are projected. Uniform wearing can be intrinsic to a greater good, or instrumental in reaching other goals. With this in mind, what data exist on the socio-political factors that influence uniform garment design and policy?

Uniform design and policy slowly changes alongside social and educational policy developments. Thus, New Zealand, uniform design has changed alongside New Zealand’s education policy and socio-political context [81]. Similarly, in China uniform has gradually incorporated more modern and Western influences in design over time [91]. In their discussion on the reasons for uniform, Meadmore and Symes argue that uniform wearing is a form of governmentality–the process of unconscious internalization of external values designed to maintain existing power structures. In this way uniform is a “disciplinary tactic” [115] embodying respectability, cleanliness, modesty, and inoffensiveness. Conformity means meeting the standards of an institution [92], explicitly in service of an ideal of equality, and implicitly to maintain the societal power dynamics expressed through institutions. Whether a form of governmentality or not, it is clear that uniform is associated with broader societal values.

In some societies, uniform wearing seems intrinsically linked to a greater societal good. Thus, Baumann and Kriskova [21] argue that high PISA scores are associated with good classroom discipline, which is intrinsically linked to wider societal values. The authors hypothesize that in South Korea and Japan, Confucian values of self-discipline and conformity to ritual inform practical aspects of daily life. Baumann and Kriskova argue that conforming to social norms is part of being a good Confucian; thus, any penalty for breaching uniform standards (a social norm) is explicitly and intrinsically linked to becoming a better Confucian.

Alternatively, uniform wearing can be instrumental in reaching other ends. Hence, when uniform use became common in the Anglosphere in the 1800’s, there seems to have been a (noble) aim of making schools islands of fairness in an unfair world. Craik [93] states that in England school uniform aimed to equalize social class, creating social camouflage through functional, reasonably priced clothing. However, this rationale ignores wider societal power structures, and that uniform wearing may be mainly instrumental to another goal. Thus, in some post-colonial contexts uniform was part of a transfer of British values and seen as a way to civilize and promote a certain ideology [92]. In New Zealand, uniforms were inspired by military dress and were intended to encourage empowerment, belonging, and pride, as well as social camouflage [92]. In South Africa, school uniforms were imposed on the black population as a means of control [36]. Australian authors have hypothesized that certain types of school uniform historically represented respectability and happiness and promoted social integration. Wearing a school uniform provided a means for migrant children (and their families) to fit in [94]. Wearing a school blazer has been described as a cultural symbol of reaching and being included in a social ideal of wealth and educational achievement [95].

Some socio-political rationales are explicit and are part of clear public policy measures to shape society. For instance, Mujiburrahaman [96] describes uniform as part of Sharia law implementation in schools in Aceh; Moser notes it is part of fostering citizenship and identity in Indonesia’s schools [97]; and Draper et al. [98] describe how uniforms that use a hybrid of traditional and modern clothing styles, materials, and manufacturing techniques are part of a cultural revitalization project in Thailand. In the United States, from the mid-1990’s school uniforms have been explicitly promoted as a means to lower danger and violence in schools and remove classroom distractions [99]. Indeed, in the United States uniforms are often perceived as more neutral than dress codes because everyone wears the same [100], as opposed to judgements being made about clothing items against a standard. Overall it appears that uniform use is often driven by goals beyond health or education as values in themselves.

### Part 3: Human Rights and Uniform Use

Human rights legislation supporting equity and freedom from religious or gender discrimination and protecting the rights of children has been discussed in conjunction with school uniform. In cases of disagreement about garment design or uniform policy and where institutional policy or social norms do not provide a solution, human rights law has been invoked to help reconcile different rights and values.

Human rights are overarching, universal entitlements that preserve the dignity of humans. Theoretically, human rights are interrelated and indivisible and should not be separated from each other [101]. Practically, the experience with uniform shows that simultaneously giving effect to different human rights is not straightforward. Social context influences how human rights are interpreted and given legal standing. Looking at the United States, Ahrens [102] notes that in the 1970’s uniform was of great constitutional concern (impinging of First Amendment right of freedom of expression), whereas nowadays few legal or constitutional problems with uniform are discussed, possibly because the overwhelming concern is student safety; the importance of identifying intruders outweighs concern over freedom of expression [103].

#### Equality vs. Equity

The human rights notion that all humans are equal is important to school uniform policy. As noted earlier, the idea that equality of access to education is enhanced by “social camouflage” is a principal historic and current rationale for uniform [36, 89]. Proponents of uniform argue it creates equality and emphasize the benefits of homogeneity that outweigh any negative impacts: unity, a sense of belonging (although this point has not been demonstrated empirically), and group identity. In their view, the human right to equal treatment is enhanced by removing outward signs of social differences [36, 89]. This may explain why in Malaysia, Woo et al. found that while students thought uniform unattractive, they conceded it reduced outward markers of differing socio-economic status [73].

However, an equality focus in uniform policy sidesteps the issue of who bears the brunt of equality as “sameness”. Equality focuses on same treatment, while equity focuses on outcomes, sometimes requiring different treatment to achieve similar outcomes [104]. Data show that uniforms are not intrinsically equitable. The cost of uniforms can affect students’ rights to access education. In addition to inequity of physical activity by gender and barriers for minority groups, the cost of uniform garments themselves is a determinant of access to education, and clearly unequally felt across society. The cost barrier that uniform poses to attending school is widespread, particularly in low and middle-income countries. Using Mongolia as an example, Sabic-el Rayess et al, [83] note that in countries where the very poor cannot afford uniforms, they do not attend school. Likewise, Simmons-Zuilkowski [105] found that in South Africa enrollment rates among the very poor are lower because of cost of uniforms. In Kenya, Mutengi [106] found a statistically significant link between uniform cost and education access, and Green et al. [107], Sitieni and Pillay [108] and Cho et al. [109] describe free uniform as part of support and incentive packages for at-risk children to attend school [110]. In Ghana, Alagbela [111] and Akaguri [112] show that uniform cost creates a barrier to education for the very poor. One contradiction to this trend comes from Hidalgo et al. in one study in Ecuador [113]. The authors found that providing uniform decreased attendance. However, the authors note that the study was not conducted as anticipated; some families promised uniforms were not supplied with them, and many in the study group had already purchased a uniform (it was therefore a sunk cost), so uniform cost was not a factor that decided school attendance. Cost is also a likely concern among all parents in high-income countries. In the United Kingdom, Davies [114] examined uniform cost and supply and surveyed parents who were happiest when uniform could be sourced from a mixture of designated shops and high street/generic stores and found that uniforms were cheapest when items could be brought from anywhere. However, as in low income countries, uniform creates an unequal cost burden across the population. In the United States, Da Costa [35] highlights the economic burden on the poor of buying a school uniform. In South Korea and the United States, poorer parents spend a higher percentage of their income on uniforms [36]. In New Zealand, a survey of parents [115] found school uniform cost is a significant burden for poorer families. In Scotland, Naven et al. [116] reported how uniform cost created such a barrier to education that the state changed its clothing grant policy to help ease the financial burden on families.

Of course cost is not the only equity issue in uniform use, but it is an important one. Davies’ [114] United Kingdom report on uniform supply and cost found that garment quality was a main influence on purchasing decisions, followed by availability and cost. Surveying parents’ and educators’ attitudes to uniforms, for both groups Davies found uniforms were considered worthwhile because they are a long-term investment: generally long-lasting, infrequently replaced, and cheaper over the student’s career than non-uniform alternatives. However, Davies’ and other data (e.g., Gasson et al., Naven et al., Catherine and Mugalavai, Simmons-Zuilkowski) suggest the large initial upfront cost is a barrier for poorer families. Another reason for concern is that sameness does not result in equity or improve human rights protection. Deane [117] argues that justifications for uniform based on equity are not well considered because the mere wearing of uniform does not create equity, and does not magic away other differences [117]. In practical terms, equity through uniforms is inevitably an imperfect idea: even if uniform policy allows students to choose to wear any items from a list so long as items comply with style or color rules, expensive branded items, or other garment choices would inevitably signal differences in economic status, wearer style, and individual preferences. It seems for the very poor/marginalized in any society, uniform can be simply another barrier to education because of the focus on equality, not equity. Ironically, those most in need of education may be denied it *via* a mechanism that was originally instituted to remove barriers to education.

#### Uniform and Freedom of Religion

In addition to general rights to equal treatment, specifically protected rights are of concern when considering uniform, particularly freedom of religion and the right to non-discrimination because of gender. Uniform rules and the right to freedom of religion is an example of where courts are asked to reconcile seemingly conflicting rights with each other. For instance, the Convention on the Rights of the Child (Art 14) protects freedom of religion [118]. Nonetheless, this right is not unfettered and can be limited if others’ rights are impinged, and its application depends on how individual countries legislate to support human rights.

Theoretically, uniforms should not impinge on religious freedom. Practically, the situation is not so clear-cut. Complex questions about how religion is represented and how it is recognized are often played out through uniform [119], especially in liberal democracies. For some, adhering to a school uniform policy means not observing religious requirements. In Australia, where states are required to have a uniform policy, direct and indirect discrimination on the basis of religion is forbidden. Yet there is no clarity on whether a school can have a policy that is silent on students’ religious beliefs and practices [120, 121]. Australian courts have found that exceptions to uniform rules can be made to avoid injury to religious sensibilities, doctrines, beliefs, or principles (e.g., allowing wearing yarmulke or hijab). In England (which has a longstanding uniform tradition), the case of *Begum* sought to balance religious freedom to wear Sharia-appropriate clothes against the right to education, school uniform policy [122, 123], and women’s rights. In *Begum* the court found that social cohesion, protecting minority rights, and ensuring religious freedom must be balanced [1, 124–126. In *Begum*, the judgment shows how tricky it is to reconcile all human rights in themselves, let alone apply them within the context uniform policy requirements.

Whatever the social context, outward signs of faith can challenge both uniform rules and wider societal values such as secularity in public institutions. Gereluk [72] argues for reasonable accommodation and mechanisms to redress potential unequal treatment of minorities. What constitutes “reasonable accommodation” appears to be context-dependent.

#### Uniform and Gender

Similarly to promoting equity and freedom of religion, human rights protect non-discrimination by gender. The discussion so far has shown that whatever the rationale, uniform garment design has a greater impact on girls, particularly on their physical health. This differential effect has been addressed by human rights legislation. For instance, The New Zealand Human Rights Commission agreed with a complaint of discrimination on gender grounds by two female-identified students [127] who argued that the requirement to wear a skirt disadvantaged them because it restricted their movement. Settlement was reached when the school added culottes (shorts that look like skirts) to the school uniform. In this example, human rights legislation allowed schools to have uniform codes for males and females, providing uniforms do not disadvantage one gender or group.

Differential treatment by gender is underpinned by historical and some current thought, though it is rarely discussed in relation to uniform. This is possibly because it is linked to deeply entrenched and normalized gender roles. Political and philosophical research addresses this point. Dussel [128] argues that school uniforms hamper, restrain, and try to domesticate girls’ bodies. Happel [87] argues that school uniform is linked to gendered performance, where school uniforms underpin sex and gender roles, because they restrict movement and confirm traditional gender identities. Happel [87] argues that because skirts allow for exposure of underwear, buttocks, and genitals, girls are taught modesty/immodesty through a garment. Girls are thus objectified because they have to curb their behavior because of another’s gaze. In this review no evidence was found of any of the above restrictions caused by boys’ uniform. Notably, girls’ uniforms tend to be more expensive [106, 114], illustrating that even here there is a “pink tax” for female-oriented products that perform the same function as a unisex/male alternative [114, 129]. Further, normalized gender roles affect gender-diverse students, already a group at risk of exclusion. For gender diverse students, non-inclusive uniform policies are particularly problematic [130] and affect them disproportionately [17]. Non-inclusive uniform policy relies upon the idea that clothing is an essential element of gender identity and that any fluidity or flexibility in dress rules risks undermining individual and collective gender identity. There is no evidence of gender identity being so fragile [131]. In practical terms, Henebery [132] argues that even if uniforms have unisex options, they are still split by gender, where skirts are limited to biological girls. Interestingly, Bragg [133] notes that a school uniform policy that strictly enforces male/female uniforms is in stark contrast with the broader and more fluid social understanding and representations of gender that students are exposed to, especially in Western countries.

It appears that uniforms place a physical restriction and price premium on girls, and policy does not routinely consider gender diverse students. This is driven by socio-cultural norms and negatively impinges on their human rights, despite the overarching right to equal treatment irrespective of gender.

#### Uniform and Children and Young People’s Freedom of Expression

Freedom of expression is another area of human rights that often clashes with uniform. The right to freedom of expression (Art 13 UNCRC [134]) can be restricted in respect of the rights and reputations of others, protection of national security, and public order. Article 12 (UNCRC, 1989) details that free expression is given weight in accordance with the age and development of the child. Some hold that school uniforms are inherently restrictive, arguing that school uniform hampers expressive rights and normal identity exploration, constitutes intrusive control of group behavior (e.g., 35), and symbolizes oppression [131]. Conversely, others argue argues that it is nonsensical to say that uniforms crush self-expression when there are many other creative outlets [89]. There is no empirical evidence on this point. Vopat takes a different approach and considers children’s moral and psychological development. Looking at expression and developmental stage, Vopat [40] separates self-expression into two categories: mere expression, and substantive expression. Mere expression is simply about what a person likes/dislikes, whereas substantive expression is an outer manifestation of deeply held values or another specific intention. Vopat [40] argues that small children lack the cognitive ability for substantive expression because they do not have the psychological capacity for it yet. Nonetheless, Vopat [40] suggests that uniform may be a learning point for students. Children need thinking time to become their moral selves. School uniforms provide explicit teachable moments, opportunities to think using different moral frameworks to examine the utility of different social attire and freedom of expression in context, and children’s understanding of and critical thinking about social appropriateness of dress [135], which enhances learning outcomes [40]. Conversely, and despite these learning opportunities, Deane [117] argues that uniform’s blindness to or suppression of difference implicitly dampens the ability think about and discuss difference; thought is constrained because uniform creates an implicit understanding that strangers should be the same as oneself, and where there is difference, there is danger. Consequently, uniform suppresses recognition and discussion about differences in ethnicity, religion, or class [117].

There is no empirical evidence either way that uniform constrains freedom of expression. There are hypotheses that uniform provides a teaching opportunity about appropriate dress, and socializes people to a particular dress standard. Other ideas suggest that uniform allows students to rebel in safe confines [81].

#### Children’s Rights and Minors as a Vulnerable Group

The rights of children sit alongside other rights. These rights protect children because the wider socio-political climate identifies children and minors as a vulnerable class of people who need protection.

However, there is no agreement about what rights of children exactly should be protected, and many wider concerns about children are projected onto uniform [89]. Through an institution limiting clothing choice or requiring certain clothing, Bodine [89] argues that uniform protects childhood by protecting children from sending messages with their clothing choices that they do not fully understand. However, exactly what is protected is unclear. Vopat [40] argues protection should be linked to the child’s moral development and ability to reason, balanced against Article 12 of UNCROC, which includes the duty to consider children’s voice in decisions that affect them. Some [87] argue that uniform should be done away with altogether because of harm to children’s human rights. Irrespective of children’s vulnerability and human rights, Brunsma and Rockquemore [136] argue that even if uniforms do not harm, and young children cannot yet exercise their rights, there is no justification for imposing uniforms in an educational context, especially if uniforms do not improve educational goals.

Overall, while human rights are universal, the way they are expressed in particular cultural contexts varies, driven by socio-political forces. It appears that the idea that uniform is inherently equitable is flawed. It does not level social class, and is not blind to religion, gender, and socio-economic status. It does not necessarily consider cultural and individual identity or diversity. Data on human rights and uniform show that uniform policies result in unequal impact of garment design and policy on girls and religious minorities. Data on freedom of expression is equivocal. Whatever the case, wider sociocultural issues are clearly played out through uniforms, and it appears that uniforms can become a proxy for other issues, particularly considering the special status of children and young people. Blanket approaches to uniform policy can be repressive of cultural identity/diversity and ignore entrenched power imbalances [22, 131]. By scrutinizing the outcomes of uniform policy, it is clear that many uniform policies have neutral/minimal impact for the majority, but the minority must compromise cultural or religious values to comply with uniform rules. Females make up half the population, yet uniform design limits their ability to participate in incidental physical activity, a proven enhancer of health and educational outcomes.

## Discussion

This review demonstrates that far from being a “trivial relic” [22], school uniform is an important yet neglected public health issue that affects all students who are required to wear it. As a preliminary review, this study maps the conceptual landscape of school uniform garment design and policy in a public health framework, and brings evidence together to show health and education impacts of school uniform use. The review shows that school uniform is important, but not for commonly believed reasons. First, there persists a belief that school uniform *in itself* enhances academic outcomes. This is unsupported by evidence—there is no direct link between uniform and academic achievement [33]. However, uniform does contribute to a more settled classroom environment [21], which facilitates learning. Second, some studies argue uniform can distract from a good rapport between students and teachers, which is linked to improved learning (30,37). Third, despite common belief, uniform has no empirically supported impact on enhancing a feeling of belonging to a school [85]. Notably, there is a general paucity of evidence for use and a gap between what is believed about uniform and what is supported by empirical evidence. It appears that uniform use and policy is a neglected area of research: given its widespread use there is surprisingly little empirical evidence about its use or effects at all.

Concerningly, psychological and physical health impacts of uniform have been neglected. Positively, uniform removes the psycho-social barrier of competitive dressing. Indeed, well-designed uniform garments that are comfortable to wear, do not restrict physical activity for all students, that protect against environmental hazards, plus a uniform policy that is inclusive of all students (irrespective of gender/gender identity) can enhance student physical and psychological health [47, 48, 54]. Neutrally, uniform can both increase and decrease bullying. Negatively, inflexible uniform policies and garment design disadvantage girls, gender-diverse students, and overweight students because they do not feel confident in participating in physical activity while wearing uniform garments (47–51,53). From a physical health perspective, empirical evidence demonstrates that girls’ physical health is particularly disadvantaged. Girls make up around half the school-aged population, so the demonstrated link between poor uniform design and worse physical and psycho-social health for girls is of concern. Physically restrictive uniforms can hamper girls’ physical and social participation in school, especially physical activity during breaks and on the journey to school. Poorly designed sports uniform may also deter girls’ and overweight children’s participation in timetabled physical education. For all students, there is no evidence of systematic consideration in uniform policy of health and safety and protection from environmental hazards that permits students to wear garments to suit the weather conditions, or that ensures garments are comfortable to wear.

Further, gender-based inequity is inherent in uniform; girls’ uniforms are more expensive and more restrictive. Inequity exists for religious minorities and gender-diverse students who have to dress to fit the uniform policy rather than dress so they feel physically comfortable. Because garment design reflects the norms of the dominant culture, religious and ethnic minorities, and gender-diverse students often have to compromise beliefs and identity to comply with uniform rules.

This review shows that uniform garment design and policy focus on equality (same treatment) at the expense of equity (different treatment to achieve similar outcomes). While uniform removes the psycho-social pressure on individuals and families of competitive dressing and outward signs of socio-economic differences between students, it does not eliminate inequity. Paradoxically, uniforms can worsen inequity. Worldwide, for the very poorest students, the cost of a uniform may be prohibitive, creating a barrier to education before the students even arrive on school grounds [83, 105–107, 109–112, 114–116, 137]. For some students the disadvantages will be cumulative. Using the public health lens of analysis highlights this avoidable inequity.

Why do we compel children to wear uniforms and persist with policies that detract from physical and psycho-social health, and that disadvantage poorer students? This review has highlighted that uniform has become a proxy for many issues. Financial and political economies are projected onto uniform policy and garment design. An organisation’s history, institutional stewardship, values, and traditions are often embodied in uniform, which is possibly why certain designs and materials are so enduring. Uniform signals a school’s place in the education market and gives external and internal indications of the school culture (22, 26, 36). Uniform also appears to enhance school operations (21). In classrooms it helps students settle to task and help identify intruders and improve security (36,43), or the perception of security (44).

A public health lens helps to shed light on uniforms, and their impact on health and education. The public health frame of analysis brings together and organizes data from different disciplines to illuminate questions that are important to population health, illustrating proximate factors and distal factors to individual experiences. It has also shown that uniform merits public health interest: if uniform use is prevalent, its use impacts on health and educational outcomes, and, importantly, school uniform garments and policies regulating their use are amenable to improvement, with an eye to improving equity.

This study’s principal limitation is that data is only drawn from English-language research largely focused on the Anglosphere or where articles were available in English, yet much of the world that wears uniform is not Anglophone. Potentially important data may have been missed. Further this study’s primary data are primarily peer-reviewed articles, which ensures rigor, but leaves out a depth of information from other sources. Further, articles of all types (including commentaries) were included because this research focused on evidence about uniform use, rather than the quality of that evidence. For time constraints conference proceedings and PhD theses were excluded. Note that there were variations in the types of studies done. For instance, the physical impacts of uniform use (e.g., on physical activity of wearers, protection against environmental hazards) were measured using quantitative or qualitative/quantitative mixes of design with larger sample sizes. For instance Norrish et al’s [48] work on physical activity for girls was one of the few that included objective and subjective measures of the phenomena under investigation, with a repeated measures crossover design (same group tested in two different conditions). Finally, as with other areas of inquiry, philosophical pieces or commentaries often argue against the status quo rather than defend it. It is possible that there exist more positive or neutral impacts of uniform on education and health than have been hitherto documented, especially in empirical research.

Limitations notwithstanding, this research will be of interest to those within the public health community, those involved in uniform regulation and design, and those involved in educational management. It will also be of special interest to the general public, who will be better informed about the evidence for what uniform achieves, and what can be done about making it better. Conceptually, issues related to uniform design are of interest to researchers of other populations (e.g., prisoners, military) with diminished capacity or whose choice of clothing is restricted.

This review has important implications for future research. It has highlighted gaps in knowledge about garment design and uniform policy and their impacts.

Regarding garment design, more information is required on different priorities that inform design choices: durability, serviceability, safety of materials, quality, and comfort to the wearer, particularly with an eye to protection against environmental hazards, and how to make garment styles enduring over time as well as inclusive, comfortable, and health-promoting.

Other issues like cost, value for money, environmental sustainability and ethical sourcing of materials may be of interest. Furthermore, different stakeholder (student, parent, teacher, school administrator) perspectives could be measured to further explore what factors influence garment design, how those different factors inform uniform use policy within schools, extending on multi stakeholder studies similar to that done by Wilken and van Aardt [42] or McCarthy et al [41]. Regarding uniform use policy, there is little information about how school rules are developed and what principles might look like to ensure uniform use is education and health promoting. Regarding impacts of design and policy, further studies are required with objective and subjective measures of whatever phenomenon related to uniform is being investigated. In particular, more studies are required on the health and psycho-social impacts of uniforms. For instance studies such as Hopkins [51], Norrish et al. [48] and Watson et al. [49] could be replicated in other jurisdictions and cultural settings.

In terms of public policy, there is little peer-reviewed evidence on supply chains, competition law, and profits that drive uniform costs. There is little evidence about how to reduce the cost barrier of uniforms for the poor; how different societal values are incorporated into uniform design (e.g., environmental protection and school/community tradition, or, given the impacts of uniform on health and access to education, whether any form of government regulation of upfront cost, uniform policy or garment design is required (especially for state-funded schools).

An important practical implication is making the evidence about uniform’s education and health impacts available in a form easily accessible to school administrators and governors to inform their uniform garment and policy decisions. After all, educators are experts in education, not garment design or uniform policy development, so it is unsurprizing that, left alone to organize uniform, they may not develop the most health and education-promoting garments or policies.

### Conclusion

Uniform use is deceptively simple. It is so commonplace and ordinary, however, the questions it sparks are complex and are related to deeply held views of what is normal, traditional, and socially acceptable. Yet uniform use has real impacts on health and education, for better and for worse. This review shows that uniforms may be the right diagnosis for creating an equitable learning environment, providing cost-effective garments over a student’s learning career, and easing the psychological pressure of competitive dressing. However, this review shows the importance of getting the prescription right. The efficacy and effectiveness of uniforms as a vehicle for equitable access to education and good health depends on the right prescription for uniform policy and garment design that remove potential negative effects of poor garment design and policy.

A public health lens reveals that much school uniform garment design and use policy negatively affects the poor, girls, religious and ethnic minorities, and gender-diverse students. It is a sad irony that these are the very groups who could benefit most from the equitable access to education that uniform is supposed to facilitate. This review also shows how environmental hazards, health and safety concerns, and garment comfort are neglected for all uniform wearers. There is no natural reason why any of this should be so.

Fortunately, any negative educational and health impacts of school uniform garment design and policy are amenable to change. The clarity that this review provides about the evidence for uniform’s impact on health and education may provide a starting point to ensure uniform is as healthy and education-promoting as possible and to build on the advantages uniform offers. By examining evidence of how uniform and uniform policy impacts on students’ health and wellbeing, perhaps it will be easier to establish a common idea about school uniform’s purpose(s), with a view to improving wearer experience. If the educational and health impacts of uniform are clear it could be possible to improve wearer experience to ensure that garments are desirable, equitable, healthy, and safe [22], and that both policies and garments enable all students to learn and thrive in modern life.
