# Corrigendum: Reviewing School Uniform Through a Public Health Lens: Evidence About the Impacts of School Uniform on Education and Health

**DOI:** 10.3389/phrs.2021.1604543

**Published:** 2021-12-13

**Authors:** Johanna Reidy

**Affiliations:** Department of Public Health, Wellington School of Medicine, University of Otago, Wellington, New Zealand

**Keywords:** school uniform, public health, equity, health impacts, education impacts, human rights

In the original article, there was a mistake in Table 2 as published. “Health and Safety can be enhanced by uniform design” should have been placed in the “Positive impact” column, not the “Neutral impact” column. “Non-inclusive design can reduce girls’ and overweight students’ confidence to participate in sport; Bullying and social exclusion for uniform following rules; Inflexible uniform policy harmful for gender-diverse students.” was placed in the “Neutral impact” column, and should be in the “Negative impact” column. In all parts of the table semi-colons were incorrectly changed to full stops. The corrected Table 2 appears below.

**TABLE 2:** Uniform’s positive, neutral, and negative impacts on education and health outcomes.

Domain	Positive impact	Neutral impact	Negative impact
Educational outcome	Improves classroom management; faster settling to task; reduced distractions	No clear impact on academic achievement	Detracts/distracts from teacher–student rapport; Reduces creativity in population; An extra barrier to socialisation for newcomers/minorities
Health outcomes: Physical	Insecticide-treated uniform can provide protection against dengue; Well-designed uniform can protect skin from sun damage; Health and safety can be enhanced in uniform design		Poor uniform policy a barrier to incidental and curriculum based exercise; Poor uniform design a barrier for incidental exercise, especially for girls; Sun protection not considered in policy or garment design; Physical comfort/health not prioritised in design; Health and safety can be misapplied to ban certain non-uniform items
Health outcomes: Psychosocial	Unisex and inclusive design can increase girls’ and overweight students’ confidence to participate in sport; Self-esteem promoted (if student can afford full and correct uniform); Removes competitive dressing pressure; Decreases bullying		Non-inclusive design can reduce girls’ and overweight students’ confidence to participate in sport; Bullying and social exclusion for following uniform rules; Inflexible uniform policy harmful for gender-diverse students

In the original article, there was an error. “and socio-cultural contexts which inform uniform use” was incorrectly written as “and socio-cultural context in which inform uniform is used.”

A correction has been made to **Results**, first paragraph, final sentence:

“Here, evidence has been arranged according to a public health lens of analysis. First, this section examines the proximate educational and health impacts of uniform garments and uniform policy on students to determine whether there are immediate health or education impacts of uniform use or policy. Second, rationales for uniform use are examined, as well as distal factors that influence student experience. This section examines the broader institutional, and socio-cultural contexts which inform uniform use.”

In the original article, there was an error. The word “is” was missing.

A correction has been made to **Part 1: Literature for Educational and Health Impacts of Uniform; Does Uniform Influence Educational Outcomes?** First sentence:

“Starting with the evidence for the impact of uniform on educational outcomes (the core in **Figure 1**), there is little convincing evidence that uniform improves academic achievement.”

In the original article, there was an error. The word “as” was missing.

A correction has been made to **Part 3: Human Rights and Uniform Use; Uniform and Freedom of Religion**, paragraph 3, first sentence:

“Whatever the social context, outward signs of faith can challenge both uniform rules and wider societal values such as secularity in public institutions.”

In the original article, there was an error. The word “has” was missing.

A correction has been made to **Discussion**, paragraph 5, second sentence:

“This review has highlighted that uniform has become a proxy for many issues.”

In the original article, there was an error. The word “it” was used in place of the word “if”.

A correction has been made to **Discussion**, paragraph 6, final sentence:

“It has also shown that uniform merits public health interest: if uniform use is prevalent, its use impacts on health and educational outcomes, and, importantly, school uniform garments and policies regulating their use are amenable to improvement, with an eye to improving equity.”

In the original article, there was an error. The word “use” was incorrectly placed after the parenthesis.

A correction has been made to **Discussion**, paragraph 7, sentence 7:

“For instance, the physical impacts of uniform use (e.g., on physical activity of wearers, protection against environmental hazards) were measured using quantitative or qualitative/quantitative mixes of design with larger sample sizes.”

In the original article, there was an error. “has school uniform” was incorrectly included in a sentence.

A correction has been made to **Conclusion**, third sentence:

“Yet uniform use has real impacts on health and education, for better and for worse.”

The author apologizes for these errors and states that this does not change the scientific conclusions of the article in any way. The original article has been updated.

